# Effects of branched-chain amino acids on changes in body composition during the recovery period following tonsillectomy

**DOI:** 10.1007/s00405-024-08902-8

**Published:** 2024-09-06

**Authors:** Réka Fritz, Ágnes Kiricsi, Miklós Csanády, Péter Fritz

**Affiliations:** 1 Doctoral School of Clinical Medicine, University of Szeged, Szeged, Hungary; 2 Department of Oto-Rhino-Laryngology and Head-and Neck Surgery, University of Szeged, Tisza Lajos Krt. 111, Szeged, 6725 Hungary; 3 Károli Gáspár University of the Reformed Church in Hungary, Faculty of Humanities and Social Sciences, Budapest, Hungary

**Keywords:** Body composition, InBody, Tonsillectomy, Body weight, BCAA

## Abstract

**Purpose:**

In recent decades studies have examined body weight changes following tonsillectomy. In nutrition science, the focus has shifted from body mass index to body composition analysis. However, no studies have explored body composition changes post-tonsillectomy. In oncology and digestive surgeries, the potential benefits of branched-chain amino acids (BCAAs) have been investigated; however, their effects on pharyngeal surgery remain unknown. Therefore, the aim of the present study was to investigate the body composition changes after tonsillectomy and to explore the potential benefits of branched-chain amino acids.

**Methods:**

This prospective interventional controlled study enrolled 48 patients who were randomly assigned to a control group (CG) and an experimental group (EG). These groups were further divided into active and inactive subgroups on the basis of their activity levels. The EG consumed 2 × 4 mg of BCAA daily. Body composition was measured using bioimpedance (InBody 270) on the day of surgery and again on days 7 and 21 postoperatively.

**Results:**

Both groups experienced similar weight loss; however, significant differences in body composition emerged. The CG showed significant muscle mass loss (from 30,29 to 28,51 kg), whereas active EG members maintained muscle mass (from 35,33 to 35,40 kg); inactive EG members increased muscle mass (from 26,70 to 27,56 kg) and reduced body fat percentage (from 31.94% to 29.87%). The general health status (InBody score) remained stable or improved in the EG (from 75,13 to 75,96); however, it decreased in the CG (from 75,42 to 72,67).

**Conclusion:**

The negative effects of tonsillectomy on body composition are mitigated by BCAA supplementation*.*

## Introduction

Tonsillectomy, as the most frequently performed otolaryngologic surgery [1], affects the first part of the digestive tract. During the recovery period of 14–21 days, patients’ eating habits considerably change owing to the significant swallowing difficulties [2]. Consequently, postoperatively, patients eat significantly less than preoperatively. During wound healing, physical work or exercise are also contraindicated to avoid bleeding [2].

Patients typically lose an average of 2.27 kg of body weight and return to their preoperative weight in approximately 5 months [3]. Moreover, following a tonsillectomy, no changes in body weight are expected 1 or 2 years postoperatively [4].

As muscle loss can occur within 10 days in critical states, swallowing difficulties and inactivity following tonsillectomy may lead to muscle wasting [5].

Body mass index (BMI), which is based on the weight-to-height ratio, is common in medical literature but misleading as it does not differentiate between fats and muscles [6]. Thus, an athlete with high muscle mass may have the same BMI as an individual with obesity of similar height and weight [7].

Recently, body composition measurement tools have been increasingly used for health assessment. In our study, we used an InBody device, which works on the principle of bioimpedance known for its ability to test body composition in different body parts (trunk and limbs) [7].

In sports nutrition, minimizing muscle loss during recovery is crucial [8]. Branched-chain amino acid (BCAA) supplements, initially used in sports, are now employed in clinical settings to support muscle maintenance during stress, including in liver disease [9–11], gastric surgery [12, 13], oncology treatments [14], and renal [15] or septic pathologies [16, 17].

However, no studies on body composition changes or the effects of BCAA following tonsillectomy have been conducted. Therefore, the aim of the present study was to investigate the body composition changes after tonsillectomy and to explore the potential benefits of branched-chain amino acids.

## Materials and methods

This prospective interventional controlled study, which was conducted from September to December 2023, included 48 participants and adhered to ethical guidelines. This study was approved by the Regional and Institutional Committee of Science and Research Ethics and the Hungarian Medical Research Council (OGYÉI/56432-2/2023) and registered on Clinical Trials (NCT06247436). Participants included those aged 20–40 years who were scheduled for tonsillectomy. Those with metabolic or bleeding disorders were excluded.

All participants underwent extracapsular tonsillectomy using the “cold knife” technique, which involves dissecting the tonsil and its capsule from the surrounding tissues using Cooper scissors, rasp, bipolar forceps, and bindings, if necessary. The inferior pole of the tonsil is removed using a snare [2].

Body composition measurements and blood tests were performed three times: preoperatively, on day 7, and on day 21 postoperatively. The wound is completely re-epithelialized at the latest 3 weeks postoperatively; therefore, the second check-up is frequently scheduled between the 14th and 21st postoperative day. We opted for the last data collection on day 21.

The following were the elements of the blood test: total cholesterol, high-density lipoprotein, low-density lipoprotein, gamma-glutamyl transferase, glutamic oxaloacetic transaminase, glutamate pyruvate transaminase, triglyceride, carbamide, creatinine, estimated glomerular filtration rate, albumin, sodium, potassium, magnesium, and calcium.

Body composition was analyzed using the InBody 270 (InBody Co., Ltd., South Korea) device, which operates on the principle of bioimpedance and is a noninvasive, fast, and accurate body composition analyzer that provides detailed information on body weight, muscle, fat mass, and water content. During the measurement, the device sends alternating low- and high-frequency electrical currents throughout the body, using the body’s water content as a conductive medium. These electrical currents are passed through electrodes in contact with the skin of the palms of the hands and soles of the feet to measure the impedance of various tissues [7].

The following were the parameters measured: weight (kg), total body water (L), body fat mass (kg), skeletal muscle mass (kg), percent body fat (%), visceral fat level, mineral mass (kg), bone mineral content (kg), protein mass (kg), basal metabolic rate (kcal), and total fitness (InBody Score). The device stores patient measurements for tracking changes over time.

Postoperatively, the study group took 2 × 4 g of BCAA powder daily. The BCAA product contained leucine, isoleucine, and valine in a ratio of 2:1:1.

Physical activity habits were recorded, and patients were divided into subgroups on the basis of whether they exercised at least three times a week.

The characteristics of the study participants are presented in Table 1.

**Table 1:** Characteristics of participants in the study

	Control			Experimental			Total		
	Inactive	Active	Total	Inactive	Active	Total	Inactive	Active	Total
N									
Female	12	4	16	11	2	13	23	6	29
Male	2	6	8	4	7	11	6	13	19
Total	14	10	24	15	9	24	29	19	48
Average age									
Female	30,7	26,5	29,6	31,8	25,5	30,8	31,2	26,2	30,2
Male	32,5	24,2	26,3	23,5	24,6	24,2	26,5	24,4	25,1
Total	30,9	25,1	28,5	29,6	24,8	27,8	30,2	24,9	28,1
SD									
Female	6,11	6,56	6,28	5,93	2,12	5,94	5,92	5,19	6,05
Male	6,36	5,71	6,63	1	7,32	5,72	5,5	6,36	6,03
Total	5,93	5,82	6,46	6,31	6,4	6,65	6,06	5,93	6,49
Average weight									
Female	65,1	61,8	64,2	67	54,1	65	66	59,2	64,6
Male	93,2	93,8	93,6	81,7	84,8	83,7	85,5	89	87,9
Total	69,1	81	74	70,9	78	73,6	70	79,6	73,8
SD									
Female	11,44	7,99	10,53	6,93	4,17	8,06	9,39	7,6	9,35
Male	9,12	11,3	10,16	16,31	9,51	11,69	14,54	10,95	11,89
Total	14,87	19,1	17,43	11,69	15,94	13,56	13,11	17,25	15,45

*C* is control group and *E* is experimental group, *I* is inactive and *A* is active, *F* is for female and *M* for men

### Statistical methods

Two- and three-variate analysis of variance (ANOVA) was used to examine the correlations between the results of the three time points and the grouping variables (test/control and active/inactive), univariate and repeated ANOVA to determine the simple main effect, and the Chi-square test to examine the deviation of the individual laboratory results from the reference range.

The prerequisites for the tests were examined using the following tests: the Shapiro–Wilk test for normality, the boxplot test for examining extreme values, Levene’s test for testing the equality of standard deviations, Mauchly’s test for testing the equality of variances (sphericity), Greenhouse–Geisser correction when the sphericity was damaged, partial η^2^ (eta) for measuring the effects of relationships that were significant following ANOVA, and Cramer’s V coefficient for measuring the effects of significant relationships following the Chi-square test.

All statistical analyses were performed using Statistical Package for the Social Sciences (version 25, IBM, Armonk, NY, USA).

## Results

The results of the two-way ANOVA with repeated measures for the three time points are shown in Table 2.

**Table 2:** Results of two-way repeated-measures analysis of variance for three time points (the day of surgery, a week after surgery, and two weeks after surgery), and groups (control and experimental) and activity (phisically active and physically inactive). The table reports results of the two-way ANOVA, including F-values, degrees of freedom (df), error degrees of freedom (df Error), p-values, and partial eta squared (η^2^), to evaluate the main and interaction effects of the independent variables on the dependent variable, along with their statistical significance and effect sizes

		F	df	df (error (time))	p	η^2^
Body weight	Time × groups	1.049	2	88	0.355	0.023
	Time × groups × activity	0.646	2	88	< 0,0005	0.429
BMI	Time × groups	0.576	1.985	87.33	0.563	0.013
	Time × groups × activity	0.199	1.985	88	< 0,0005	0.276
SMM/body weight ratio	Time × groups	41.795	2	88	< 0,0005	0.487
	Time × groups × activity	9.672	2	88	0.001	0.156
BFM/body weight ratio	Time × groups	16.782	2	88	< 0,0005	0.276
	Time × groups × activity	0.997	2	88	< 0,0005	0.186
Visceral fat level	Time × groups	8.434	1.966	86.523	< 0,0005	0.161
	Time × groups × activity	8.135	1.966	85.017	< 0,0005	0.416
Total body water/body weight ratio	Time × groups	8.133	2	88	0.001	0.156
	Time × groups × activity	1.547	2	0	0.526	0.014
InBody Score	Time × groups	31.32	1.932	85.017	< 0,0005	0.416
	Time × groups × activity	1.952	1.932	88	< 0,0005	0.487

**Fig. 1:**
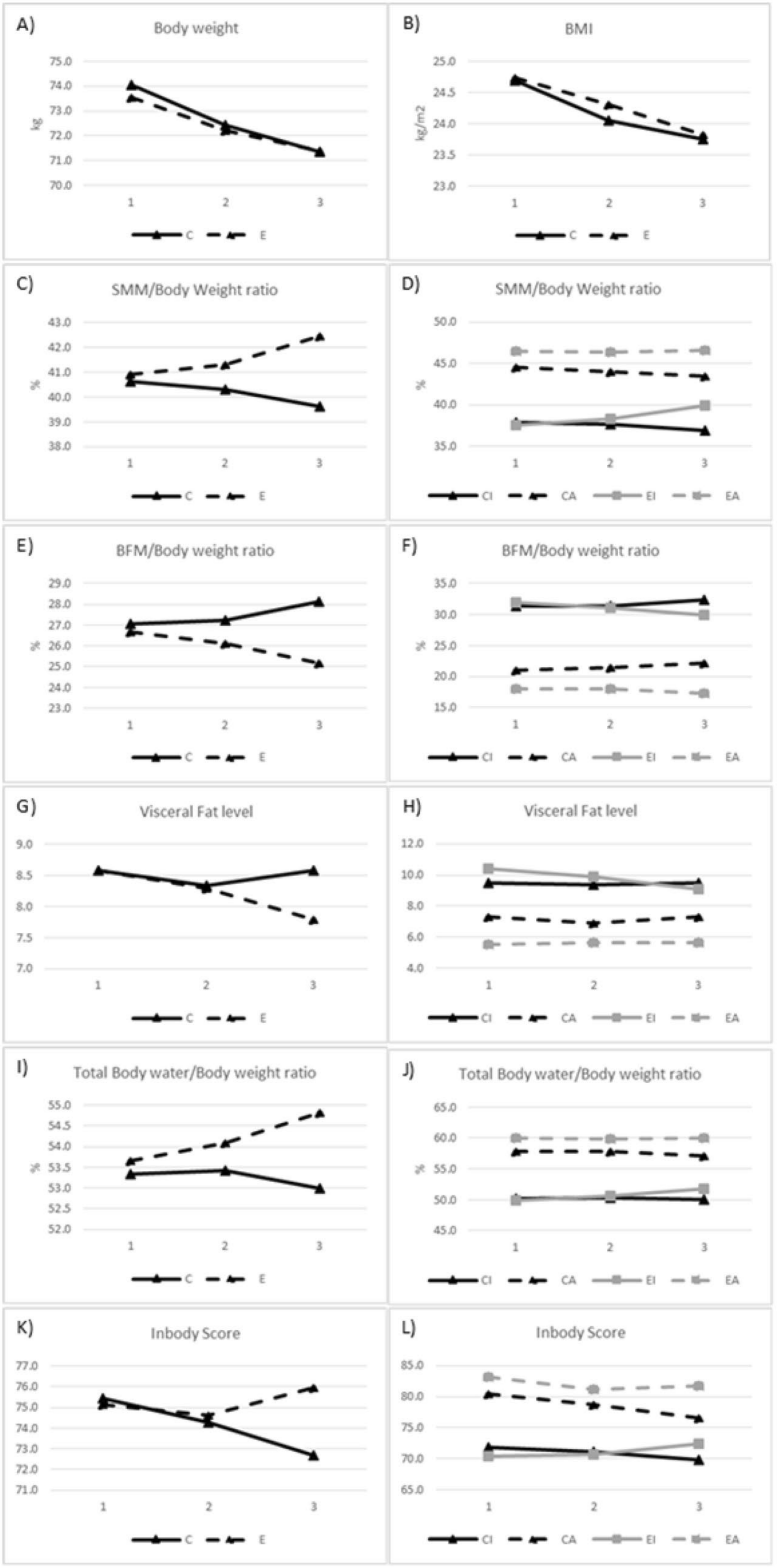
Comparison of changes in body weight (**A**), BMI (**B**), SMM/body weight ratio (**C** and **D**), BFM/body weight ratio (**E** and **F**), visceral fat level (**G** and **H**), total body water/body weight ratio (**I** and **J**), and InBody Score (**K** and **L**) between the control (**C**) and experimental (**E**) groups and the *CI* control inactive, *CA* control active, *EI* experimental, inactive, and *EA* experimental active groups; for the three time points: the day of surgery (1), 1 week postoperatively (2), and 2 weeks postoperatively (3)

The body composition test results are depicted in Fig. 1.

A two-variable ANOVA was performed to understand the effects of BCAA on the different elements of body composition postoperatively. Furthermore, a three-variable ANOVA was performed to determine the potential influence of exercise habits.

We examined the conditions for the tests. Body composition data, except for SMM and TBW, were normally distributed (p > 0.05) according to the Shapiro–Wilk test. Moreover, examining the boxplots showed no extreme values in the data. Homogeneity of standard deviations (by group) existed for all body composition items and time points, with the exception of one case (InBody Score at time point 2), as determined by Levene’s test for the equality of standard deviations.

The equality of variances (sphericity) was performed using Mauchly’s sphericity test. The Greenhouse–Geisser correction was used in the interaction analysis when the equality of variances was violated.

We presented the results of the seven body composition elements on the basis of the prerequisite test. For the body composition results, we presented the interaction of the results of the test and control groups measured at three time points, and when a significant three-way relationship with exercise habits was demonstrated, their results were also presented.

The mean body weight continuously decreased in both groups and less in the study group; however, no significant (p = 0.341) interaction was noted between the changes in body weight of patients who took supplements and those who did not.

Regarding BMI, no significant interaction (p = 0.565) was observed between the two groups and the values measured at the three time points.

A significant and strong two-way interaction was demonstrated between the two groups in terms of the muscle mass-to-body weight ratio (F [1.458, 64.164] = 41.795, p < 0.0005, partial η2 = 0.487). A continuous increase in the ratio of muscle mass to body weight was observed in the patients of the study group, whereas those in the control group exhibited a continuous decrease. A significant and strong interaction was noted between the changes in the muscle mass-to-body weight ratio, dietary supplement consumption, and exercise habits (F [1.458, 64.164] = 9.672, p = 0.001, partial η^2^ = 0.180). In the study group, a significant increase was observed in the inactives, stagnation in the actives in the study group, and a decrease in both inactives and actives in the control group.

A significant and strong two-way interaction was demonstrated between the changes in the body fat-to-body weight ratio over time and dietary supplement consumption (F [1.461, 64.272] = 16.782, p < 0.0005, partial η_2_ = 0.276). The percentage of body fat significantly decreased in the study group, whereas it significantly increased in the control group. The influencing factor for exercise could not be detected using the three-variable ANOVA (p = 0.352). In the study group, both actives and inactives lost weight, whereas the body fat percentage increased in both cases in the control group.

A two- and three-way significant interaction was noted between the measured value, dietary supplement, and effects of sport in terms of the visceral fat level. The two-sided correlation was significant, and the degree of interaction was high (F = [2, 88] = 8.434, p < 0.0005, partial η^2^ = 0.161). The visceral fat level decreased in both groups at the second examination time point; however, it decreased further at the third examination time point. Additionally, an increasing rate was noted among the participants in the study group, whereas the direction of change was reversed among those in the control group, and it increased to a level above the initial value.

The three-way significant relationship was also strong. (F(2, 88) = 8.135, p = 0.001, partial η^2^ = 0.156). In this case, the actives in the study group were stagnated, the inactives in the study group continued to decrease, and both actives and inactives in the control group initially experienced a decrease and a subsequent increase to the original value.

The interaction between the body water-to-body weight ratio and dietary supplement consumption was strong and significant (F = [1.501, 66.061] = 8.133, p = 0.002, partial η^2^ = 0.156). The percentage of body water increased in those who consumed BCAA, whereas an average increase of 0.1% was observed in the control group at the first time point, followed by a decrease. Exercise had no effect (the three-way interaction was not significant, p = 0.222). Regardless of the sporting lifestyle, the body water percentage value increased in the participants in the study group, whereas it decreased in those in the control group.

The InBody Score reflects a general assessment of the body composition. Using this score, a significant two-way interaction was observed in relation to dietary supplement consumption (F = [2, 88] = 31.320, p < 0.0005, partial η2 = 0.416). At the second measurement time point, the value decreased in both groups; however, at the third time point, the measured value of the control group continued to decrease, whereas it increased above the original value in the study group. No significant three-way interaction was observed (p = 0.148). However, among the actives in the study group, the value increased following the initial decrease, whereas among the inactives, a continuous increase was noted. In both cases, a continuous decrease was observed in the control group.

Regarding body composition test results wherein a significant two-way interaction was noted, we also examined the simple main effects to understand the extent and direction of the individual factors (control/test and time) influencing the dependent variable, independent of the other factor.

No significant main effects were observed between the control and study groups in any case (for any of the body composition indicators and for any of the time points); however, a significant change over time was noted in all cases.

Regarding laboratory results, the deviation from the reference range was measured using the Chi-square test, whereas the strength of the relationship was measured using Cramer’s V coefficient.

Regarding blood test results, significant changes in the examined elements were note noted. Neither dietary supplement consumption nor the grouping based on lifestyle activity revealed any differences between the groups.

## Discussion

This study aimed to evaluate the changes in body composition in adult patients in the recovery period following tonsillectomy.

Analyzing body composition instead of relying on BMI and body weight, which have been the most commonly used methods for nutritional status assessment, is a novel concept. Individuals with the same age, height, and weight (therefore, the same BMI [kg/m^2^]) can have different body shapes, body composition, energy requirements, and metabolic profiles [18].

Regarding tonsillectomy, and pharyngeal surgeries in general, there are no published studies in the literature that focus on body composition.

The muscle is the body’s primary glycogen store; during critical periods such as the recovery period following tonsillectomy, energy is primarily sourced from muscles rather than fats [19].

In this prospective interventional controlled study, we introduced the use of BCAA to our study group to prevent muscle mass degradation. This approach ensured that the energy requirement was met by utilizing fat, thereby preserving muscle mass.

BCAAs including leucine, isoleucine, and valine are unique in sports nutrition as muscles can directly use them without liver involvement. They are quickly digested, providing energy and preserving muscle mass, thereby contributing to their anticatabolic effect [20], as confirmed in our study.

No clear dosage recommendation for BCAAs is reported in the literature. Depending on the state of health and individual goals, a 1–60 g daily supplement may even be effective [21].

In the literature, some researchers examined the effects of this dietary supplement on the body using 2 × 6 g of BCAA daily [22], others used 2 × 14 g daily [23], whereas another group used 6 g of BCAA daily [24].

Considering these different data and the fact that the patients in our study are in a strict physical rest period, but also in the wound healing phase, which puts a strain on the body, we decided on 8 mg (2 × 4 mg) BCAA daily.

Considering patients’ physical activity habits is the strength of the present study, as active individuals generally have higher muscle mass. Notably, our active patients had an initial muscle mass of 36.2 kg, which was significantly higher than the 26.4 kg of the inactive group, consistent with literature findings.

This approach allowed us to identify significant differences not only between the control and study groups but also between physically active and inactive patients.

Moreover, the most valuable component of our research results is that we examined not only the changes in specific body composition elements within each group but also the changes in these parameters compared with patients’ body weight. Comparing these values to each other and expressing them in proportion for each patient helps to eliminate the possibility of error caused by potentially large anthropometric differences between individual patients.

We examined the elements determined using the InBody 270 that can significantly change over the 3-week follow-up period (body weight, skeletal muscle mass, percentage body fat, visceral fat, total body water, and InBody Score). Additionally, the impact of BCAA, a dietary supplement that protects muscles and promotes muscle synthesis, was assessed.

Our study aligns with existing literature on tonsillectomy and body weight, showing no novel findings. We observed a 2.67 kg weight loss in the control group and 2.15 kg in the study group, similar to the reported 2.27 kg loss in adults following tonsillectomy [3].

However, solely focusing on body weight can be generally misleading; body composition data support the significance of our findings.

We briefly summarize the most significant results of our study as follows:

Despite the low-impact lifestyle, patients in the study group showed a continuous increase in muscle mass (from an average of 30.3 to 30.5 kg) and a parallel decrease in body weight (from an average of 73,55 to 71,40 kg), with a consequential decrease in fat percentage (from 26.69% to 25.15%). Patients in the control group showed a continuous decrease in muscle mass (from 30.3 to 28.5 kg) and body weight (from 74,02 kg to 71,35 kg), with a consequential increase in fat percentage (from 27.06% to 28.13%) in both subgroups.

In the active study group, the average muscle mass practically did not decrease, whereas the muscle mass of the inactive study group increased. Although this group also lost body weight, its muscle mass was maintained such that this group met the body’s energy requirements mainly from fat. The same explanation accounts for the changes in visceral fat observed in our study.

In the nutritional point of view, muscle mass represents the qualitative aspect of “body weight,” being the key of fitness status of the human body [8].

Our results indicated that the BCAA dose used in this study effectively protected muscle mass in the active group with relatively higher muscle mass and promoted muscle synthesis in the inactive group with lower initial muscle mass. In contrast, the muscle mass of the control group decreased in the absence of the dietary supplement. In this case, patients’ energy needs were primarily covered by muscle breakdown.

The literature has indicated that the skeletal muscle mass-to-body water ratio in body composition tests is directly proportional [25]. The physiological explanation for this finding lies in the ability of muscle mass to bind water. Therefore, when a loss of muscle mass occurs, a consequent decrease in body water percentage can also be observed.

Conversely, an increase in muscle mass is also expected to result in an increase in body water.

Consistent with these data in our study, the percentage of body water, which is similar to muscle mass, increased in the study group, whereas it decreased in the control group.

The InBody Score, also termed the fitness score, integrates various metrics, including muscle mass, body fat percentage, and body water. A higher muscle mass is frequently correlated with improved body composition and reduced fat percentage, positively influencing the InBody Score [26, 27]. In our study, the changes in the InBody Score for each group matched the changes in muscle mass and body fat.

No significant changes were observed in liver or kidney function or ion levels, confirming the safety of BCAA supplementation.

Our study suggests that BCAA is beneficial during tonsillectomy recovery, preventing muscle loss and supporting a quicker return to normal activity.

Nowadays, patient satisfaction and quality of life play an increasingly significant role in patient care. Based on the results of our study, recovery with BCAA supplementation is associated with a higher state of fitness, thereby warranting its inclusion in postoperative treatment and care protocols.

This study had some limitations that should be acknowledged. First, stronger results may be achieved with a larger sample size. To mitigate this limitation, we not only examined changes in individual body composition components over time but also their ratios to current body weight. Second, the differences would have been more pronounced if we had examined the differences between professional athletes and average individuals as a measure of physical activity; this could be the goal of a future research.

For future perspectives, BCAA supplementation could be beneficial for patients with more serious pharyngeal diseases, such as cancer. Maintaining optimal general health is crucial for responding to treatments and mitigating the side effects of oncological therapies in these patients.

## Data Availability

The data that support the findings of this study are not openly available due to reasons of sensitivity and are available from the corresponding author upon reasonable request.
